# Expanding the *HPSE2* Genotypic Spectrum in Urofacial Syndrome, A Disease Featuring a Peripheral Neuropathy of the Urinary Bladder

**DOI:** 10.3389/fgene.2022.896125

**Published:** 2022-06-23

**Authors:** Glenda M. Beaman, Filipa M. Lopes, Aybike Hofmann, Wolfgang Roesch, Martin Promm, Emilia K. Bijlsma, Chirag Patel, Aykut Akinci, Berk Burgu, Jeroen Knijnenburg, Gladys Ho, Christina Aufschlaeger, Sylvia Dathe, Marie Antoinette Voelckel, Monika Cohen, Wyatt W. Yue, Helen M. Stuart, Edward A. Mckenzie, Mark Elvin, Neil A. Roberts, Adrian S. Woolf, William G. Newman

**Affiliations:** ^1^ Manchester Centre for Genomic Medicine, Manchester University NHS Foundation Trust, Manchester, United Kingdom; ^2^ Division of Evolution, Infection, and Genomics, Faculty of Biology, Medicine, and Human Sciences, University of Manchester, Manchester, United Kingdom; ^3^ Division of Cell Matrix Biology and Regenerative Medicine, School of Biological Sciences, Faculty of Biology Medicine and Health, University of Manchester, Manchester, United Kingdom; ^4^ Department of Pediatric Urology, KUNO Clinic St. Hedwig Clinic, University Medical Center Regensburg, Regensburg, Germany; ^5^ Department of Clinical Genetics, Leiden University Medical Centre, Leiden, Netherlands; ^6^ Genetic Health Queensland, Royal Brisbane and Women’s Hospital, Herston, QLD, Australia; ^7^ Department of Pediatric Urology, Ankara University School of Medicine, Cebeci Children’s Hospital, Ankara, Turkey; ^8^ Sydney Genome Diagnostics, Children’s Hospital at Westmead, Westmead, NSW, Australia; ^9^ Disciplines of Child and Adolescent Health and Genomic Medicine, University of Sydney, Sydney, NSW, Australia; ^10^ Städtisches Klinikum Dessau, Dessau-Roslau, Germany; ^11^ Department of Medical Genetics, Hospital La Timone, Marseille, France; ^12^ Center for Human Genetics and Laboratory Diagnostics (AHC) Medical Labs Martinsried, Martinsried, Germany; ^13^ Biosciences Institute, Medical School, Newcastle University, Newcastle, United Kingdom; ^14^ Protein Expression Facility, Manchester Institute of Biotechnology, University of Manchester, Manchester, United Kingdom; ^15^ Peak Proteins Ltd., Macclesfield, United Kingdom; ^16^ Royal Manchester Children’s Hospital, Manchester University NHS Foundation Trust, Manchester Academic Health Science Centre, Manchester, United Kingdom

**Keywords:** HPSE2, urofacial, heparanase-2, LRIG2, missense, Ochoa syndrome, triplication, rare disease

## Abstract

Urofacial (also called Ochoa) syndrome (UFS) is an autosomal recessive congenital disorder of the urinary bladder featuring voiding dysfunction and a grimace upon smiling. Biallelic variants in *HPSE2*, coding for the secreted protein heparanase-2, are described in around half of families genetically studied. *Hpse2* mutant mice have aberrant bladder nerves. We sought to expand the genotypic spectrum of UFS and make insights into its pathobiology. Sanger sequencing, next generation sequencing and microarray analysis were performed in four previously unreported families with urinary tract disease and grimacing. In one, the proband had kidney failure and was homozygous for the previously described pathogenic variant c.429T>A, p.(Tyr143*). Three other families each carried a different novel *HPSE2* variant. One had homozygous triplication of exons 8 and 9; another had homozygous deletion of exon 4; and another carried a novel c.419C>G variant encoding the missense p.Pro140Arg in *trans* with c.1099-1G>A, a previously reported pathogenic splice variant. Expressing the missense heparanase-2 variant *in vitro* showed that it was secreted as normal, suggesting that 140Arg has aberrant functionality after secretion. Bladder autonomic neurons emanate from pelvic ganglia where resident neural cell bodies derive from migrating neural crest cells. We demonstrated that, in normal human embryos, neuronal precursors near the developing hindgut and lower urinary tract were positive for both heparanase-2 and leucine rich repeats and immunoglobulin like domains 2 (LRIG2). Indeed, biallelic variants of *LRIG2* have been implicated in rare UFS families. The study expands the genotypic spectrum in *HPSE2* in UFS and supports a developmental neuronal pathobiology.

## Introduction

Urofacial (Ochoa) syndrome (UFS) is rare autosomal recessive disease featuring urinary voiding dysfunction and a grimace upon smiling (Elejalde, 1979; Ochoa 2004; Newman and Woolf, 2018; Osorio et al., 2021). The urinary tract phenotype is characterized by bladder dyssynergia, with the detrusor contracting against and incompletely dilated outflow tract. This is manifest by dribbling incontinence of urine, and the residual urine is prone to bacterial infection. Moreover, high intravesical pressures lead to vesicoureteric reflux (VUR) which, if accompanied by urosepsis, can cause kidney infections, scarring and end-stage kidney failure. The characteristic grimace when smiling or laughing results from an abnormal contraction of the corners of the mouth and eyes (Ochoa 2004; Aydogdu et al., 2010).

Biallelic variants in *HPSE2*, coding for the secreted protein heparanase-2 (McKenzie et al., 2000; McKenzie 2020), was the first gene implicated in UFS (UFS1; Mendelian Inheritance in Man #236730). Indeed, *HPSE2* variants have been described in around half of the families with the syndrome who have been genetically investigated (Newman and Woolf, 2018). There is variability in phenotypic expression, even in a single family, and a small proportion of affected individuals may manifest only the grimace or urinary voiding dysfunction (Newman and Woolf, 2018). Previous reports of pathogenic variants in *HPSE2* feature stop-gain variants, splice variants and deletions (Daly et al., 2010; Pang et al., 2010; Al Badr et al., 2011; Stuart et al., 2015; Bulum et al., 2015; Vivante et al., 2017; van der Ven et al., 2018; Cesur Baltacı et al., 2021), all consistent with a loss of function mechanism. There exists only a single report of a homozygous missense, p.(Asn543Ile), in *HPSE2* associated with UFS (Mahmood et al., 2012).

In mice, heparanase-2 has been immunodetected in pelvic ganglia (Stuart et al., 2015), structures that send postganglionic autonomic neurons to the bladder (Keast et al., 2015). *Hpse2* mutant mice have dysfunctional bladders (Guo et al., 2015; Stuart et al., 2015) with impaired dilatation of the bladder outflow tract (Manak et al., 2020) and abnormal patterns of bladder nerves (Roberts et al., 2019). Moreover, experimental knockdown of *hpse2* in *Xenopus* leads to dysmorphic peripheral nerves (Robert et al., 2014). The specific biological role, or roles, of heparanase-2 are less clear but the protein has the abilities to bind heparin and heparin sulphate, and to inhibit the enzymatic (e.g., heparan sulfate degrading) activity of classical heparanase (Levy-Adam et al., 2010), here called heparanase-1, by outcompeting binding to its heparan sulfate substrate. Heparanase-2 also modulates both the migration of human tumour cells *in vitro* and experimental tumour growth *in vivo* (Gross-Cohen et al., 2021a; Gross-Cohen et al., 2021b).

Rarer individuals with classical features of UFS have biallelic variants in *LRIG2* (UFS2; Mendelian Inheritance in Man #615112), encoding a plasma membrane associated protein called leucine rich repeats and immunoglobulin like domains 2 (Stuart et al., 2013; Fadda et al., 2016; Sinha et al., 2018). Biallelic missense variants in *LRIG2* have also been reported in rare individuals with bladder dysfunction and renal failure, but who lack the facial phenotype (Roberts et al., 2019). Like heparanase-2, LRIG2 is detected in mouse pelvic ganglia (Stuart et al., 2015), and homozygous *Lrig2* mutant mice have bladder dysfunction and abnormally patterned bladder nerves (Roberts et al., 2019).

In this study, we sought to expand the *HPSE2* genotypic spectrum in families with UFS and make further insights into its pathobiology by seeking heparanase-2 and LRIG2 proteins in peripheral nerve precursors in human embryos.

## Patients and Methods

### Genetic analyses of *HPSE2* and *LRIG2*

All individuals reported in this study provided consent to participate in a study to define the genetic cause of their family diagnosis. Institutional ethical approval for the study was granted (United Kingdom; University of Manchester [06138] and National Research Ethics Service Northwest, Greater Manchester Central ethics committee [06/Q1406/52 and 11/NW/0021]). Where a clinical diagnosis of UFS was made prior to genetic testing a targeted approach of sequencing *HPSE2* and *LRIG2* was employed. Where there was clinical uncertainty, but UFS lay within the differential diagnosis a broader candidate gene or unbiased exome approach was employed. In Families 1 and 4, Sanger sequencing was undertaken for all coding exons of *HPSE2* and *LRIG2.* Primers for amplification of exons and exon-intron boundaries of *HPSE2* and *LRIG2* were designed with Primer3Plus. For *HPSE2* NM_021828.4 for exons 1 to 12 and NM_001166246.1 for transcript variant 4 alternative exon 12 (exon12b) was used (details available on request). For *LRIG2* NM_014813 for exons 1–18 was sequenced (details available on request). Sanger sequencing was performed using the BigDye Terminator v3.1 kit (Life Technologies, CA, United States) according to manufacturer’s instructions and resolved on an ABI3730 sequencer (Life Technologies, CA, United States). Genotyping for *HPSE2* and *LRIG2* variants was undertaken by sequencing the relevant amplicons in other family members. In Family 2, genetic testing was performed by a TruSight One capture kit (Illumina) using Nextera rapid capture for 4813 genes that were considered clinically relevant at the time of the design. This was sequenced on a NextSeq550 (Illumina) with 2 × 150 bp paired-end reads. The alignment to GRCh37 was performed on NextGene (SoftGenetics, v2.4.1) with the in-built copy number variation (CNV) tool for CNV detection. Bioinformatic analysis of 57 genes (Supplementary Data) associated with urinary tract malformations was performed. In Family 2, confirmation of the CNV detected and cascade testing of family members were undertaken on an Agilent SurePrint G3 Human whole genome microarray. In Family 3, a trio analysis with a CytoScan HD single nucleotide polymorphism array was undertaken (ThermoFisher Scientific). Data were processed and analysed using NxClinical v5.1 software (BioDiscovery, CA, United States). Results were confirmed visually in whole exome sequencing data. In short, capture was performed using the SureSelect Human All Exon V7 capture kit (Agilent) according to manufacturer’s instructions and subsequent sequencing was performed on a NovaSeq 6000 sequencing system (Illumina). Mapping was performed using an in-house GATK-based pipeline and resulting data was visualized using the Integrative Genomics Viewer (IGV, Broad Institute, CA, United States) (Robinson et al., 2017).

### Transfection of HPSE2 in Mammalian Cells

FreestyleTM HEK293-F cells (Thermo Scientific) were transiently transfected in FreestyleTM 293 expression medium (Thermo Scientific) in duplicate with either wild-type pcDNA3: HPSE2c myc, or the myc-tagged p.Asn543Ile (Mahmood et al., 2012) or p.Pro140Arg (current paper) variant constructs. Three days after transfection at 37°C shaking at 130 rpm, the cultures were split and heparin (10 μg/ml) was added to one set and left to grow for a further 24 h with shaking at 37°C. Heparin is known to bind wild type heparanase-2 and in cultured cells adding heparin to the media will sequester heparanase-2 protein that was associated with the cell surface (Levy-Adam et al., 2010; McKenzie, 2020). The conditioned media was clarified and concentrated 10-fold using a vivaspin 5 KDa concentrator. Ten µg of cell lysate protein per lane was used for western blotting. β-actin was used as a cellular loading control. For the supernatant lane loadings equal amounts of cells were quantified using a haemocytometer and the clarified media was collected next day for blotting. All samples were concentrated to the same volume to standardise before SDS analysis and blotting. RIPA lysis buffer was added to the cell pellets on ice and left for 30 min. The lysates were sonicated (30% setting for 30 s on ice) and then centrifuged at 15,000 g for 30 min at 4°C. Samples were removed and mixed with 2 x SDS Laemmlli buffer containing 2-mercaptoethanol and heated for ^95o^C for 5 min. Samples were resolved on a 4–20% SDS PAGE gel and blotted onto polyvinylidene fluoride membranes. Membranes were blocked in PBS-T, 5% skimmed milk for 1 h and then incubated overnight with anti-myc antibody (Sigma) at 4oC. Next day, membranes were washed with PBS-T, milk and incubated with secondary mouse anti-myc antibody for 1 h in PBS-T milk. Blots were finally washed in PBS-T and incubated with ECL reagents (GE). Chemiluminescence was detected using the Syngene western blot system.

### Immunohistochemistry

Human embryonic tissues, collected after maternal consent and with ethical approval (REC18/NE/0290), were sourced from the Medical Research Council and WellcomeTrust Human Developmental Biology Resource (https://www.hdbr.org/). Seven week embryonic tissues were fixed, paraffin embedded, and sectioned as described (Lopes et al., 2019) and serial sections were immunostained with one of the following primary antibodies: rabbit anti-heparanase-2 (1:200; custom made and raised against an epitope starting at amino acid 82) (Roberts et al., 2014); rabbit anti-LRIG2 (1:200; AP13821b; Abgent); or chicken anti-β3-tubulin (1:400; AB9354; Millipore). The primary antibodies were detected with secondary antibody and signals generated with a peroxidase-based system, as described (Lopes et al., 2019).

## Results

### Family 1

**FIGURE 1:**
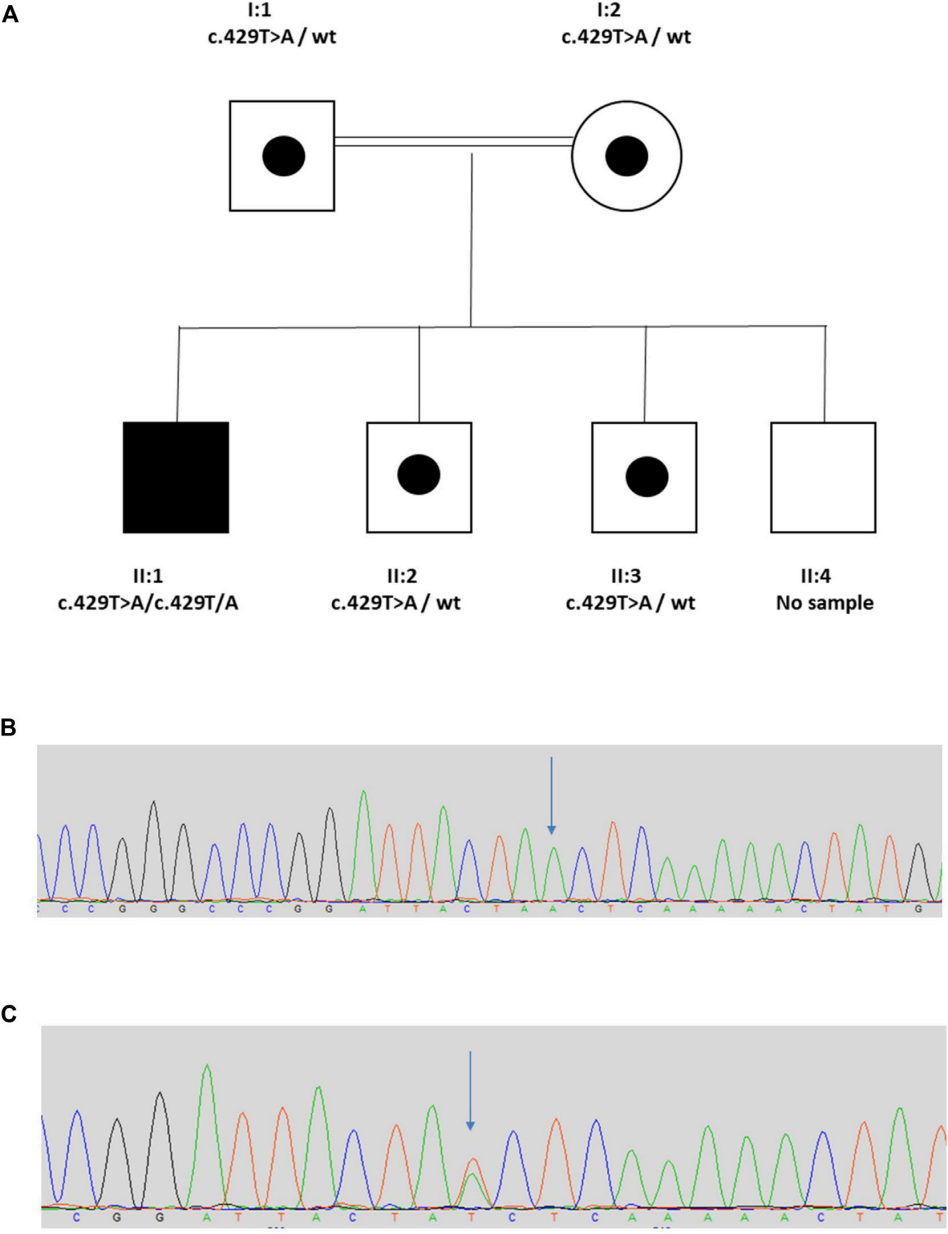
Family 1. **(A)**. Pedigree of Family 1 with individual affected with UFS (II:1) shaded. Dots represent clinically unaffected individuals confirmed to carry a *HPSE2* heterozygous variant. **(B,C)**, Genomic sequence chromatograms, showing variant c.429T>A indicated by an arrow **(B)** homozygous variant for affected, **(C)** heterozygous variant for carriers. Please see Supplementary Video 1 for the grimace upon smiling.

The proband (II:1) is a 20 year old male from a consanguineous Turkish family (Figure 1). His clinical course featured recurrent urinary tract infections (UTIs), and VUR was diagnosed when he was 7 years old. He was initially treated with intermittent bladder catheterization and anticholinergic medication. His small capacity bladder was surgically augmented when he was 12 years old. Subsequently, he reached end-stage renal failure and received a kidney transplant at the age 17 years. UTIs persisted and a video-urodynamic study demonstrated VUR into both his native kidneys and into the transplanted kidney. He has a striking grimace upon attempting to smile (Supplementary Video S1). The proband’s parents are clinically unaffected. Sequencing of *LRIG2* revealed no significant variants. Sequencing of *HPSE2* in the proband revealed a homozygous pathogenic variant, as defined under ACMG guidelines (PVS1, PM2_Mod, PS4_Supp) (Richards et al., 2015) c.429T>A, p.(Tyr143*), which has previously been reported in a family affected by UFS (Stuart et al., 2015). Segregation analysis revealed that his parents, who were each clinically unaffected, were each heterozygous for the variant, as were two of the proband’s clinically unaffected brothers (II:2 and II:3) where DNA samples were available. A sample from the other unaffected brother (II:4) was not available.

### Family 2

**FIGURE 2:**
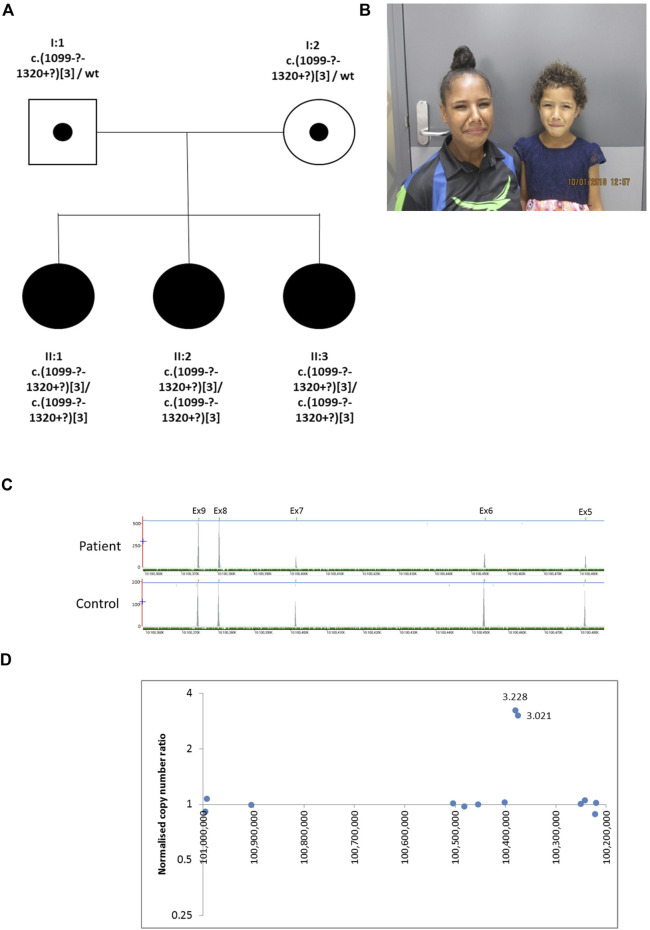
Family 2. **(A)**. Pedigree of the Family 2 with three individuals affected with UFS (shaded). Dots represent clinically unaffected individuals confirmed to carry a *HPSE2* heterozygous variant **(B)**. Grimace when smiling characteristic of UFS in individuals II:2 and II:3. **(C)**. Y-axis indicates raw depth of coverage after alignment from NGS gene panel; X-axis chromosomal coordinates in chr10 (Note that *HPSE2* is on the minus strand). The blue line indicates the gene *HPSE2* and the green dashes the exons within *HPSE2* Exon numbering according to NM_021828.4. **(D)** Copy number analysis in *HPSE2*: Normalised copy number ratio of patient against sex-matched controls. Data points for captured regions within *HPSE2*. Y-axis shows relative copy number against control samples, where 1 indicates having normal diploidy; X-axis chromosomal coordinates on chromosome 10 in reverse orientation. Two consecutive exons in *HPSE2* showed high normalised copy number ratio indicative of homozygous triplication (total of six copies).

The proband (II:2), is the middle of three sisters born to a non-consanguineous couple (Figure 2) from the Torres Strait Islands, Australia. The pregnancy leading to her birth, and the birth itself, were uneventful. She has a grimace typical of UFS. She presented aged 3 years with recurrent UTIs. Investigations revealed that she had a thickened bladder wall, VUR and hydronephrosis. She had a bladder stoma fashioned at that point. She had an ileocystoplasty bladder augmentation in her early teenage years and she currently self-catheterises. Investigations in her early teenage years revealed persistent thickening of the bladder wall, with bladder diverticulae and a large residual volume after micturition, together with VUR, bilateral hydronephrosis and kidney cortical scarring. Her blood creatinine was elevated at 106 μmol/L (upper normal for age 82 μmol/L). II:3 is her younger sister who presented with a UTI aged 4 years. Investigations revealed bilateral VUR with a thickened neurogenic bladder. She currently self-catheterises. Like II:2, II:3 has a grimace typical of UFS, and she also had a bladder that failed to void completely, with a residual of 117 ml, with a thickened wall and bilateral VUR. II:3’s blood creatinine was elevated at 63 μmol/L (upper normal for age 58 μmol/L). The eldest sister, II:1, had had several UTIs as a child but did not report other urinary symptoms. Ultrasonography revealed a normal bladder capacity (205 ml) and a minimal residual volume (2 ml) after micturition, and the upper urinary tract also appeared normal. She did not have an overt grimace on attempting to smile. In the proband in Family 2 next generation sequencing of a panel of genes associated with urinary tract malformations identified a potential homozygous triplication of exons 8 and 9 in *HPSE2*, p.(Val367_Pro [3]). This variant is defined as variant of uncertain significance (4F, 5D) using the ACMG and ClinGen guidelines (Rooney Riggs et al., 2020). This was confirmed by a high resolution microarray (arr [GRCh37] 10q24.2 (100365741_100390995)x6. Segregation analysis by microarray revealed that the two sisters were also homozygous for the triplication and the parents were heterozygous. Sequencing of *LRIG2* revealed no significant variants in the proband.

### Family 3

**FIGURE 3:**
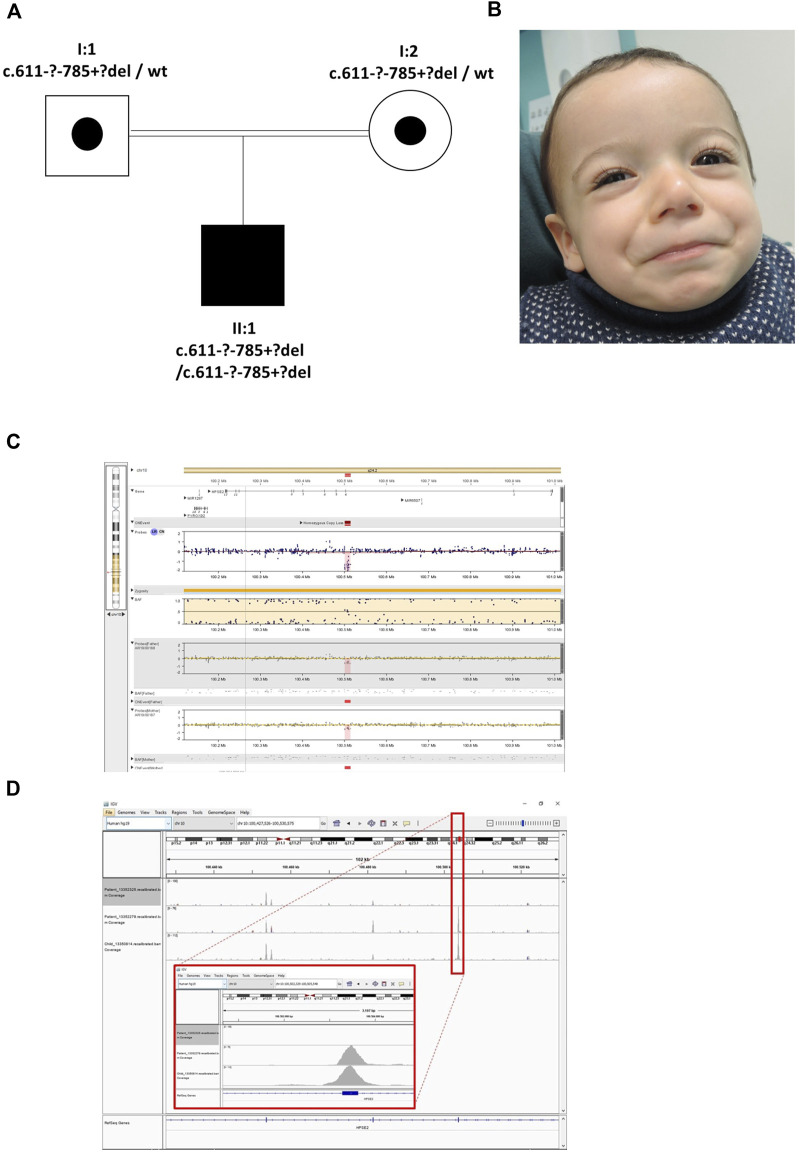
Family 3. **(A)**. Pedigree of the Family 3 with individual affected with UFS (shaded). Dots represent clinically unaffected individuals proven to carry a *HPSE2* heterozygous variant. **(B)**. Facial appearance of child demonstrating downturned corners of the mouth when smiling. **(C)**. The SNP array shows the proband (top track) with the father and mother below, both with a heterozygous deletion. The BAF (B allele frequency) panel of the picture shows a part of the region of homozygosity (marked yellow) and in the probes track the homozygous deletion depicted in pink/red. **(D)**. The IGV image shows the coverage of the WES zoomed to encompass *HPSE2* exon 4. The top track is the proband with the homozygous deletion (no coverage of exon 4). The two tracks below are two control individuals in the same WES capture/sequence run demonstrating exon coverage.

The proband was the son of consanguineous first cousin Turkish parents (Figure 3) who had been referred with a tentative diagnosis of facial nerve paresis. Otherwise, he was fit and well with no developmental problems. He was toilet trained at 3 years of age, with only occasional episodes of enuresis. There was no history of UTIs and a bladder ultrasound at 3 years of age was normal, with no residual urine volume after micturition. Later in childhood, however, there was a history of mild dribbling urinary incontinence during the day, and there was a significant volume (70–158 ml) of urine after micturition. He defecates small amounts, four to five times a day. SNP microarray of the proband revealed a potential homozygous deletion of exon 4 in *HPSE2*, arr [GRCh37] 10q24.2 (100501035_100514963)x0. with a minimal size of 13.9 kb. Subsequent exome sequencing confirmed this finding. His parents had no urinary tract symptoms or facial signs, and they were both heterozygous for the deletion. The deletion of exon 4 is in frame, leading to the predicted loss of 58 amino acids and formation of a truncated protein p.(Ala_Asn261del). This variant is defined as a variant of uncertain significance (2E, 5H) using the ACMG and ClinGen guidelines (Rooney Riggs et al., 2020). Upon further assessment, the proband’s abnormal smile, with downturned corners of the mouth upon smiling, was considered consistent with UFS. Sequencing of *LRIG2* revealed no significant variants in the proband.

### Family 4

**FIGURE 4:**
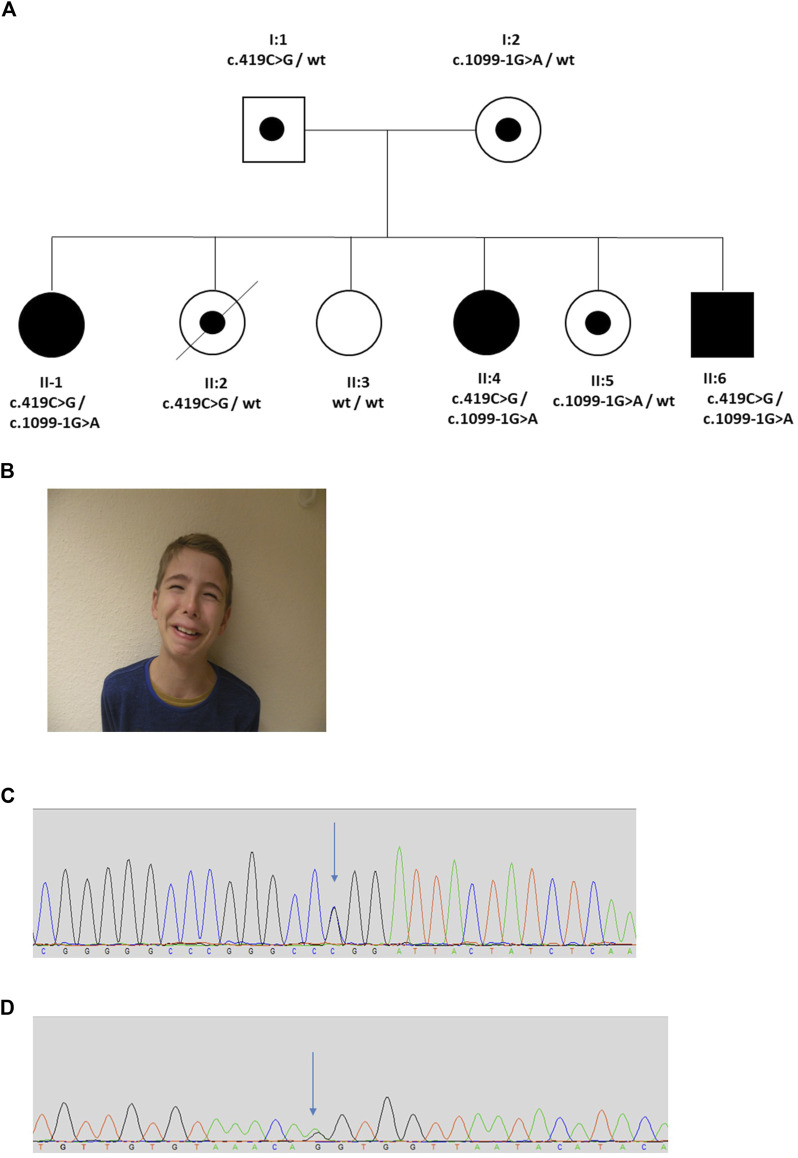
Family 4. **(A)**. Pedigree of the Family 4 with three individuals affected with UFS (shaded). Dots represent clinically unaffected individuals confirmed to carry a *HPSE2* heterozygous variant. **(B)**. Grimace when smiling characteristic of UFS in individual II:6 in the family. **(C,D)**. Genomic sequence chromatograms showing the *HPSE2* heterozygous variants indicated by an arrow **(C)** c.419C>G, and **(D)** c.1099-1G>A.

The proband (II:1) was female and the first child of healthy non-consanguineous German parents (Figure 4). She presented in early childhood with recurrent UTIs associated with poor bladder emptying and she was initially treated with intermittent bladder catheterization and anticholinergics. Magnetic resonance imaging revealed normal spinal anatomy. She underwent a vesicostomy aged 4 years and assessment in her seventh year led to a diagnosis of neurogenic bladder with detrusor sphincter dyssynergia. Her course was complicated by VUR and damage to her left kidney so that, as assessed by isotope scanning when 12 years old, it contributed only 22% of total kidney function. The next two siblings (II-2 and II-3) were also female. They had no urinary symptoms, but II-2 died at 18 years from epilepsy. The next sibling (II:4), another girl, presented *in utero* with a thickened bladder wall reported on an anomaly screening ultrasound scan. After birth, a micturating cystourethrogram (MCU) was abnormal, consistent with a neurogenic bladder. When assessed aged 17 years, urodynamics revealed a low compliance bladder with high intravesical pressures of 70 cm H_2_O after filling, rising to 180 cm H_2_O during micturition, the latter over three times the upper limit of normal (Lemack et al., 2002). MCU revealed incomplete bladder emptying with 130 ml residual urine. Ultrasonography revealed bilateral hydronephrosis and her overall renal function was impaired with a blood creatinine of 114.4 μmol/L (upper normal 105.6 μmol/L). The fifth sibling (II:5) was a healthy female. The sixth and final sibling (II:6) was a boy. He was investigated for nocturnal enuresis aged 10 years when urodynamics revealed a low compliance and low-capacity bladder with staccato micturition. Ultrasonography showed normal kidneys and blood tests showed normal renal function. He commenced intermittent self-catheterization and anticholinergic therapy. A grimace when smiling was noted and the diagnosis of UFS was considered; in further inspection the two other siblings with urinary tract disease were also noted to have a grimace. Sequencing of *LRIG2* revealed no significant variants. Sanger sequencing of *HPSE2* identified two variants, c.419C>G, p.Pro140Arg and c.1099-1G>A in the three affected children (II:1, II:4, and II:6). Two sisters unaffected by urinary disease (II:2 and II:5) were carriers for a single variant, and the third clinically unaffected sister (II:3) was wild type. The parents were heterozygous carriers, confirming that the variants were on separate alleles, consistent with autosomal recessive inheritance. The c.1099-1G>A variant has previously been reported in a patient with UFS (Stuart et al., 2015) and is predicted to result in the loss of a splice acceptor within exon 8, so introducing a premature stop codon. This variant was classified as pathogenic (PVS1, PM2_Mod, PS4_Supp, PP1_Supp, PM3_Mod) by ACMG guidelines (Richards et al., 2015; Ellard et al., 2020). Before this report, however, the c.419C>G variant has not been associated with UFS. This variant occurs at a minor allele frequency of 0.00001 (i.e., 2 in 152,996 alleles in the gnomAD database) (Karczewski et al., 2020). The c.419C>G variant is a missense change, predicted to result in the substitution of a proline for an arginine residue, p.Pro140Arg. This proline residue is conserved in heparanase-2 between humans to zebrafish. *In silico* tools of pathogenicity gave conflicting predictions: the variant was disease-causing as assessed with Mutation Taster (http://www.mutationtaster.org/); benign on Polyphen-2 (http://genetics.bwh.harvard.edu/pph2/); but tolerated using the Sorting Intolerant From Tolerant (SIFT) tool (http://sift.bii.a-star.edu.sg/); and with a Combined Annotation Dependent Depletion (CADD) score of 22.4 (a score of >20 predicts that this variant in the top 1% of most likely deleterious variants) (Rentzsch et al., 2019). This variant is classified as a VUS (PM2_Mod, PP3, PM3_Mod, PP1_Supp) according to ACMG guidelines (Richards et al., 2015; Ellard et al., 2020).

### Protein Modelling and *in vitro* Expression of Heparanase-2 Missense Variants

**FIGURE 5:**
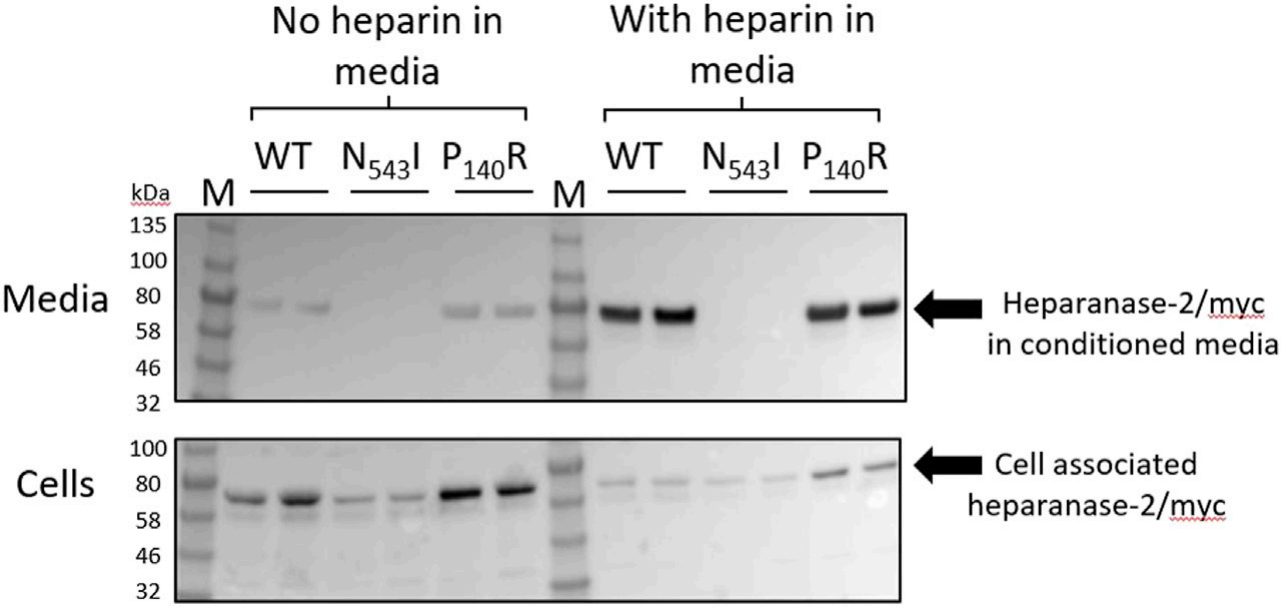
Expression of wild type and missense variant proteins in HEK293 cells. Non quantitative western blot analyses using anti-myc antibody. HEK293 cells were transfected with pCDNA3:HPSE2cmyc wild-type (*WT*) or the myc-tagged the p.Asn543Ile variant (abbreviated to *N543I* in the annotated image) or the p.Pro140Arg variant (abbreviated to P140R in the annotated image) variant. Samples, with two replicates shown for each condition, were studied at both 3 days after transfection (*No heparin in media*) and also 24 h later after the addition of heparin to the media (*With heparin in media*). The upper blot is of the conditioned media (*Heparanase-2/myc in condition media*)*,* while the lower blot is from cell lysates (*Cell associated heparanse-2/myc*). Note that all three proteins were detected in cells, and that the wild-type and the p.Pro140Arg variant were detected in the conditioned media. The lack of the p.Asn543Ile variant in conditioned media was documented both before and after adding heparin to the media; this molecule is known to bind native heparanase-2 (McKenzie, 2020). In blots of both the wild-type and the p.Pro140Arg variant, the addition of heparin to the media appeared to increase the intensities of the detected protein bands in the supernatant compared with the intensities of the bands that were cell-associated.

In order to learn more about missense variant discovered in Family 4, we undertook further analyses. First, we expressed the p.Pro140Arg variant in cultured cells, and compared it with the p.(Asn543Ile) variant previously reported in a family with UFS (Mahmood et al., 2012). Each protein was expressed as a myc-tagged protein in HEK293 cells (Figure 5). Each variant protein was detected in cell lysates, as for cells transfected with a wild-type *HPSE2* construct, also myc-tagged. As expected for a secreted protein, wild-type heparanase-2 was also detected in the conditioned media. In this context, however, there was a contrast between the two missense proteins, with only p.Pro140Arg being detected in the media. In parallel experiments, heparin, a molecule that binds wild-type heparanase-2 (McKenzie, 2020), was added to the media. Again, the myc-tagged p.Asn543Ile variant was not detected in the supernatant. In blots of both the wild-type and the p.Pro140Arg variant, the addition of heparin to the media appeared to increase the intensities of the detected protein bands in the supernatant compared with the intensities of the bands that were cell associated.

Second, we undertook protein modelling. No heparanase-2 structural information exists. The closest structural homologue is human heparanase-1 (encoded by *HPSE*) with approximately 50% sequence identity at the protein level (Wu et al., 2015). The heparanase-1 precursor is arranged into signal peptide-small subunit-proteolytic linker-large subunit. In the mature protein, the linker is cleaved off to form a ‘heterodimer’ of small and large subunits. The residue Asn543 in heparanase-2 (Mahmood et al., 2012) corresponds to Asn496 in heparanase-1 (using precursor numbering). This Asn position is strictly invariant in the heparanase-1/heparanase-2 orthologues and is located in the large subunit as part of the beta-sandwich domain. Asn496 in heparanase-1 forms main-chain hydrogen bonds with a nearby beta-strand to maintain the sandwich domain. These hydrogen bonds are likely conserved in heparanase-2, as suggested by the Alphafold predicted model (Tunyasuvunakool et al., 2021) and the p.Asn543Ile substitution in heparanase-2 is predicted to interfere with these hydrogen bonds. The effect of altering the residue Pro140 in heparanase-2 is more complex to rationalise (Supplementary Figure S1). Pro140 (underlined and cyan shaded in Supplementary Figure S1) and the surrounding residues are not well conserved with heparanase-1 in terms of sequence and structure. The equivalent region in the latter is part of the proteolytic linker (red letters) that gets cleaved off during activation to form the mature protein. It is therefore difficult to predict a possible effect of this variant in the absence of the heparanase-2 structure.

### Immunolocalisation of Heparanase-2 and LRIG2 in Normal Human Embryos

**FIGURE 6:**
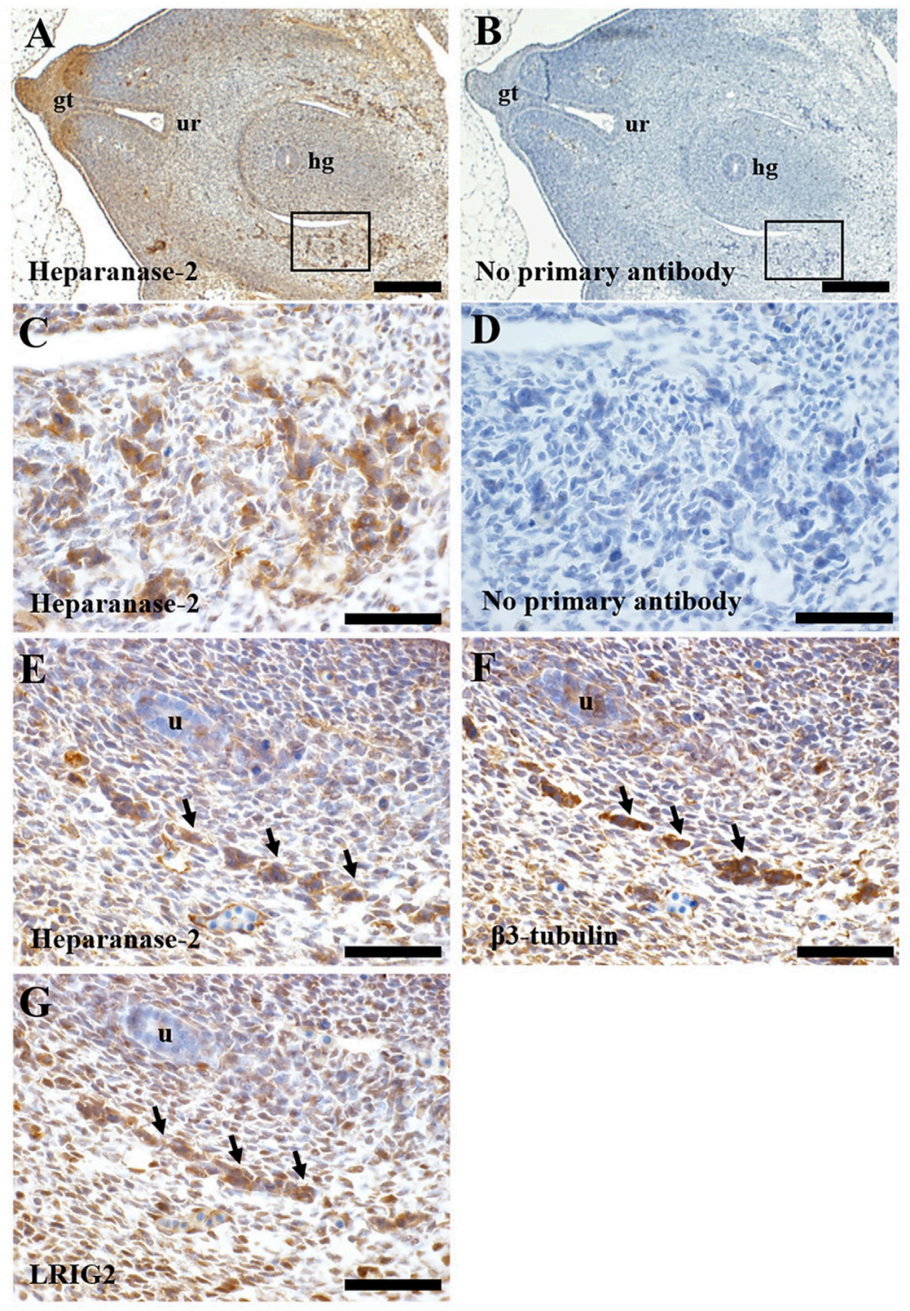
Immunohistochemistry of human embryos. Transverse sections through a seven-week human embryo at the level of the hindgut. All sections were counterstained with haematoxylin (blue nuclei). In all sections, ventral (the front of the embryo) is to the left, and dorsal is to the right. A and B are low power views, while the other frames are high power views. **(A)**. Immunostaining for heparanase-2 (brown signal). Note positive staining in collections of cells (one area is boxed) flanking the hindgut (hg). Positive immunostaining is also evident in the genital tubercle (gt) and the forming urethra (ur). **(B)**. Adjacent section with primary antibody omitted; no brown signal is detected. **(C)**. High power approximating to the boxed area in **(A)**. Note collections of cells that immunostain for heparanase-2. **(D)**. Adjacent section to that depicted in **(C)** but with primary antibody omitted. **(E–G)**. These are serial sections flanking the hindgut taken from the same embryo but more cranial to the area viewed in **(C)**. Note the cord of cells (arrowed) that are positive for each of these three proteins: heparanase-2 **(E)**; β3-tubulin **(F)**, a protein enriched in neurons; and LRIG2 **(G)**. The embryonic ureter (u) is seen in the same three sections. Scale bar is 200 µm in A and B, and 20 µm in **(C–G)**.

We studied histology sections of two human embryos, each of 7 weeks gestation. At this stage, the hindgut has separated from the urogenital sinus, the latter being the precursor of the urinary bladder (Jenkins et al., 2007). The immunostaining patterns were similar in each embryo. Heparanase-2 was immunodetected in loosely aggregated collections of cells flanking the hindgut (Figure 6), and also in the in the primitive urethra and in the genital tubercule. In transverse sections more cranial to these, heparanase-2 was detected in cords on cells flanking the hindgut and near the embryonic ureter. Serial sections revealed that these structures immunostained for the neural marker β3-tubulin as well as for LRIG2. The latter protein was also detected in other cells in this region, including loosely packed stromal-like cells.

## Discussion

Our study expands the genotypic spectrum in *HPSE2* in UFS and supports a developmental neuronal pathobiology. In a broader context, *HPSE2*-related disease can be placed among other early onset lower urinary tract dysfunctional diseases associated with variants in genes that code for other molecules involved in neural and smooth muscle maturation (Beaman et al., 2019; Houweling et al., 2019; Mann et al., 2019; Woolf et al., 2019).

**FIGURE 7:**
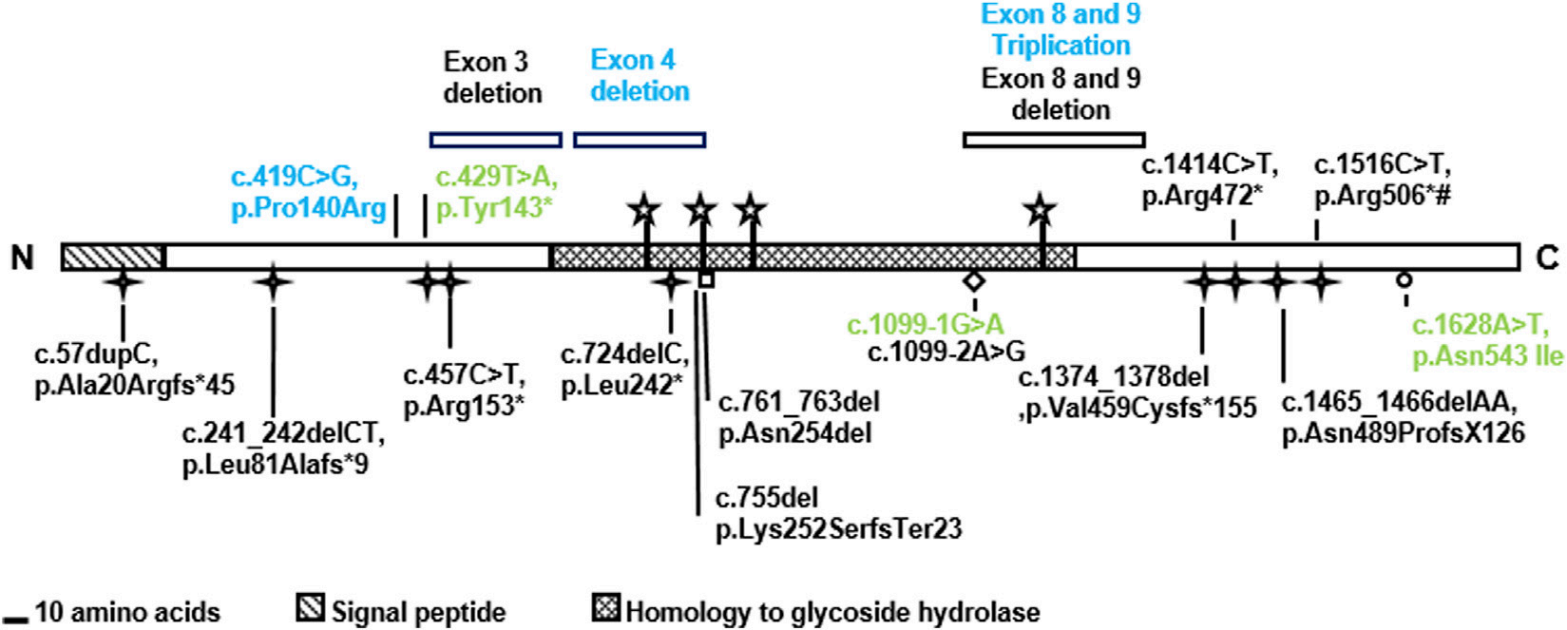
Summary of *HPSE2* variants associated with UFS. The novel variants found in the three families in the current report are shown in blue. The variants in green have been reported in previous studies. Two of them were identified in the affected individuals in this study and the missense variant p.Asn542Ile was considered for its functional consequence. Yet other variants, shown in black, have been reported in previous publications that investigated UFS.

We studied four previously unreported families with UFS carrying *HPSE2* variants. In one family, the proband had end stage kidney failure and was homozygous for the previously described pathogenic variant c.429T>A, p.(Tyr143*). The three other families each carried a different novel *HPSE2* variant. One had homozygous triplication of exons 8 and 9; another had a homozygous deletion of exon 4; and one carried a novel c.419C>G variant encoding the missense p.Pro140Arg in *trans* with c.1099-1G>A, a previously reported pathogenic splice variant. Expressing the missense heparanase-2 variant *in vitro* showed that it was secreted as normal, suggesting that 140Arg has aberrant functionality after secretion. The c.419C>G missense variant is only the second reported case in which a variant of a missense variant in *HPSE2* associated with UFS (Mahmood et al., 2012) and no previous cases of *HPSE2* exon triplication have been associated with UFS. The study therefore expands the *HPSE2* genotypic spectrum associated with UFS (Figure 7 and Table 1).

**TABLE 1:** Summary of HPSE2 variants associated with UFS. The table contains both results from historical reports as well as the current report.

*HPSE2* mutation	Predicted protein change	Reference
c.57dupC	p.(Ala20Argfs*45)	Daly et al. (2010)
c.241-242delCT	p.(Leu81Alafs*9)	Pang et al. (2010)
c.419C>G	p.Pro140Arg	Current report
c.429T>A	p.(Tyr143*)	Stuart et al. (2015)
		and current report
c.457C>T	p.(Arg153*)	Daly et al. (2010)
		Bulum et al. (2015)
		Vivante et al. (2017)
c.724delC	p.(Leu242*)	Stuart et al. (2015)
c.755del	p.(Lys252SerfsTer23)	Cesur Baltacı et al. (2021)
c.761-763del	p.(Asn254del)	Stuart et al. (2015)
c.1099-1G>A	p.(Val367Glyfs*2) or p.(Val367Lysfs*6)	Stuart et al., 2015., and current report
c.1099-2A>G	p.(Val367Glyfs*2) or p.(Val367Lysfs*6)	van der Ven et al. (2018)
c.1374-1378del	p.(Val459Cysfs*155)	Al Badr et al. (2011)
c.1414C>T	p.(Arg472*)	Daly et al. (2010)
c.1465-1466delAA	p.(Asn489Profs*126)	Daly et al. (2010)
c.1516C>T	p.(Arg506*)	Pang et al. (2010)
c.1628A>T	p.Asn543Ile	Mahmood et al. (2012)
Exon 3 deletion c.449-?-610+?del	p.(Asp150-Thr203del)	Daly et al. (2010)
Exon 4 deletion c.611-?-785+?del	p.(Ala204-Asn261del)	Current report
Exon 8-9 deletion/insertion	p.(Val367-Pro440del)	Daly et al. (2010)
c.1099–4166-1320 + 840delins23		
Exon 8–9 triplication	p.(Val367-Pro440 [3])	Current report
c.(1099-?-1320+?)[3]		

It is likely that UFS is under-reported with few individuals with urinary bladder voiding under-going genetic studies and the association between a facial grimace and bladder dysfunction not being considered (Newman and Woolf 2018). It is still surprising, however, how few missense variants in this gene have been associated with disease. Furthermore, there are many genetic mechanisms by which loss of function can arise and these new findings of copy number variation demonstrate the importance of incorporating comprehensive analysis methods in diagnostic testing when UFS is suspected. Deletion of exon 3 and a complex deletion of exons 8 and 9 in *HPSE2* have previously been reported (Daly et al., 2010; Stuart et al., 2015).

Exon (Lücking et al., 2001) or whole gene (Singleton et al., 2003) triplication is very rarely reported as a mutational mechanism in Mendelian disorders and has not previously been reported for *HPSE2*. It is interesting to note that an insertion-deletion of exons 8 and 9 in *HPSE2* has been reported associated with UFS (Daly et al., 2010). We did not define the breakpoints or exact rearrangement of the triplication and it is not possible to know if it is in frame or not and how it would impact on protein coding. If in frame, the translation would result in a significant structural change to the protein and alter its stability, whereas an out of frame change would be predicted to result in nonsense-mediated decay. Each would be consistent with the loss of function of mechanism associated with other *HPSE2* variants in UFS (Newman and Woolf 2018).

Four potential isoforms of heparanase-2 have been envisaged as a consequence of differential splicing of exons 3 and 4 (McKenzie et al., 2000; McKenzie 2020). Previously, we identified a whole exon deletion of exon 3 in a family with UFS (Daly et al., 2010). Our finding in the current report of a child with UFS with a homozygous exon 4 deletion suggests that the isoforms containing both exons 3 and 4 are critical for heparanase-2 function.

In the current study, we demonstrate that the previously reported p.Asn543Ile amino acid substitution in heparanase-2 in a family with UFS (Mahmood et al., 2012) generates a protein that apparently fails to be secreted. Hence this missense variant likely acts through a loss of function mechanism, providing experimental evidence that this a potential mechanism for other putative loss of function variants (Newman and Woolf 2018). In contrast with the p.Asn543Ile variant, the p.Pro140Arg variant reported here encodes a secreted protein. The functional effect of this variant is still to be elucidated but may result, for example, in altered interaction with heparanase-1 (Levy-Adam et al., 2010) together with, for example, disruption in potential downstream intracellular signalling (Roberts et al., 2014; Roberts et al., 2016). The variable clinical presentations in the affected individuals in Family 4 (e.g. the milder urinary tract phenotype in II:6), suggests that additional factors beyond the *HPSE2* genotype contribute to the phenotype. These co-factors include the severity, frequency and type of UTIs and modifier genotypes.

Although there are a limited number of reports of individuals with variants in *HPSE2* and *LRIG2* to draw definitive phenotype-genotype correlations it is of interest to explore if there are differences. The similarity of clinical phenotype between cases with biallelic loss of function and missense variants in *HPSE2* suggests that these missense variants result in a loss of function (Daly et al., 2010; Pang et al., 2010; Mahmood et al., 2012; Stuart et al., 2015). This contrasts with the spectrum of disease associated with *LRIG2* variants where biallelic loss of function variants result in classical UFS whereas biallelic missense variants cause severe bladder voiding dysfunction with no facial phenotype (Stuart et al., 2013; Roberts et al., 2019). This suggests that hypomorphic missense variants of *LRIG2*, resulting in reduced function or expression, have a clinical phenotype whereas there is no evidence to date that hypomorphic *HPSE2* variants result in disease. It is possible that such hypomorphic variants in *HPSE2*, if they exist, result in a different clinical phenotype.

Finally, our new observations of heparanase-2 localisation in early human embryogenesis is broadly consistent with the hypothesis that the bladder manifestations of UFS are the result of, at least in part, a peripheral neuropathy affecting the lower urinary tract (Roberts et al., 2016; Roberts and Woolf 2020). We detected both heparanase-2 and LRIG2 in neural-like cells with a migratory phenotype and these are postulated to be pelvic ganglia precursors (Keast et al., 2015). The current results complement an existing human report that, later in the first trimester, both proteins are present in nerves located between detrusor muscle bundles (Stuart et al., 2013). While the pattern of bladder nerves not been studied in native tissues of individual with UFS, it is notable that mice carrying mutations of either *Hpse2* or *Lrig2* each have bladder bodies and outflow tracts containing abnormally patterned neurons (Roberts et al., 2019). It can be postulated that heparanase-2 is required for the normal differentiation and functionality of human bladder nerves. In this context, an interaction with heparanase-1 is possible because this protein is also detected in pelvic ganglia (Stuart et al., 2015) and, at least in rat phaeochromocytoma cells, heparanase-1 modulates neuritogenesis (Cui et al., 2011). Of note, cell biology experiments implicate LRIG2 in axon guidance (van Erp et al., 2015) and in controlling cell turnover in neural tumour cells (Xiao et al., 2018). Further experiments are now required to determine the possible effects of LRIG2 on bladder nerve precursor cells.

## Data Availability

The original contributions presented in the study are included in the article/Supplementary Material, further inquiries can be directed to the corresponding authors. The data presented in the study are deposited in the CLINVAR repository, accession numbers SCV002525226-SCV002525230.
