# Supplementary figures and images for: Expanding the HPSE2 Genotypic Spectrum in Urofacial Syndrome, A Disease Featuring a Peripheral Neuropathy of the Urinary Bladder
Source: Front Genet. 2022 Jun 23;13:896125. doi: 10.3389/fgene.2022.896125 (PMC9259970; doi:10.3389/fgene.2022.896125)

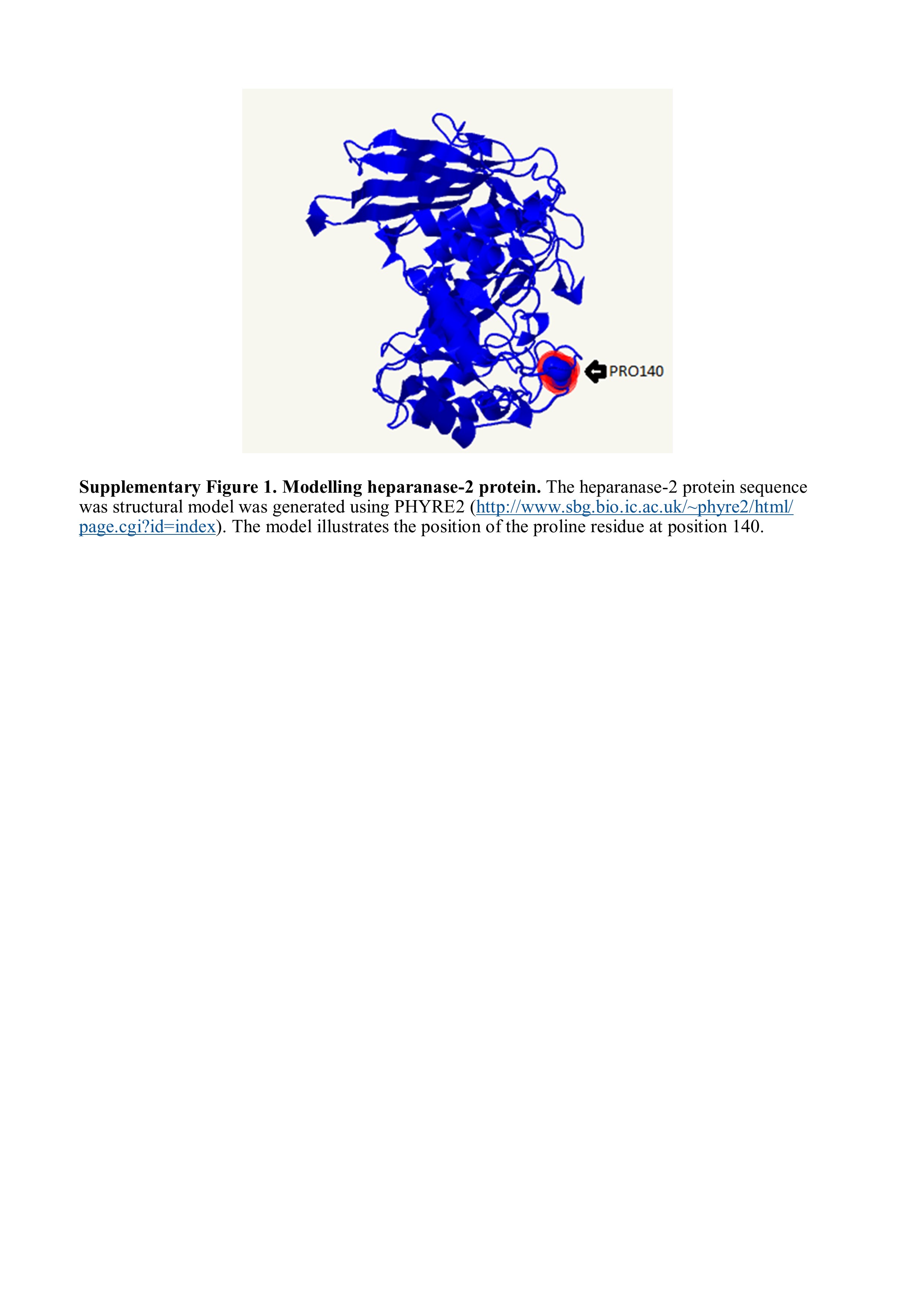

Supplement: Supplementary file 1 [file Image1.JPEG]
